# Oral pre-exposure prophylaxis implementation in South Africa: a case study of USAID-supported programs

**DOI:** 10.3389/frph.2024.1473354

**Published:** 2024-12-10

**Authors:** Jerome Wendoh Milimu, Lauren Parmley, Mahlodi Matjeng, Mathata Madibane, Mandisi Mabika, Jacques Livingston, Joseph Lawrence, Orapeleng Motlhaoleng, Hasina Subedar, Rethabile Tsekoa, Zandile Mthembu

**Affiliations:** ^1^ Bilateral Health Office, United States Agency for International Development, Pretoria, South Africa; ^2^ National Department of Health, South African Government, Pretoria, South Africa

**Keywords:** HIV prevention, pre-exposure prophylaxis, PEPFAR program, South Africa, cabotegravir, dapivirine ring, lenacapavir, health systems

## Abstract

Since the introduction of oral pre-exposure prophylaxis (PrEP) in 2016, countries have successfully scaled-up PrEP to populations at risk of HIV acquisition, including key populations, serodiscordant couples and pregnant women. Between 2016 and 2023, there were over 5.6 million oral PrEP initiations globally. Of these, over 1.2 million occurred in South Africa, with nearly 700,000 implemented through USAID/South Africa's PEPFAR program. This case study uses WHO's Building Blocks for Health Systems Strengthening to describe USAID's oral PrEP program in South Africa, reporting experiences and lessons learned in 14 districts across 7 provinces. Key lessons include: (i) Substantial donor financial investment was critical for expanding oral PrEP in South Africa, but sustained leadership and investment from government stakeholders, such as the Department of Health and the National Treasury, have been essential for sustainability. Despite fluctuations in USAID funding, annual PrEP initiations have continued to increase in USAID-supported districts largely due to local leadership. (ii) Health information and supply chain systems required agility to monitor oral PrEP introduction and scale-up. When systems lacked agility, temporary solutions like the development of interim reporting tools were necessary. (iii) Integrating community-based and facility-based service delivery supported client-centered care. Nurses and lay health workers contributed to over 80% of the full-time equivalents supporting PrEP under USAID's human resources for health portfolio. (iv) Integrating sexual and reproductive health services with oral PrEP service delivery provided clients with comprehensive, client-centered care. (v) Other client-centered care included differentiated service delivery options, such as mobile and gazebo modalities, and expanded PrEP choice through implementation science activities for new PrEP products. (vi) USAID-supported PrEP initiations have been highest among females of reproductive age in the general population and men who have sex with men among key populations, priority populations in South Africa. As done in this case study, sharing best practices and lessons learned from USAID/South Africa's oral PrEP program can strengthen the implementation evidence base and inform more efficient PrEP service delivery, particularly as new PrEP products become available.

## 1 Introduction

South Africa has the largest HIV epidemic globally with an estimated 7.8 million people living with HIV in 2023 (1). With nearly 150,000 new cases estimated in 2023, the nation still has a high rate of new infections despite advancements in testing and treatment, highlighting opportunities to strengthen HIV prevention coverage (1). Early HIV prevention efforts starting in the 1980s, led by the South African government and non-governmental organizations, focused on raising public awareness on modes of transmission (2), followed by the establishment of condom distribution programs (3). Subsequently, in the late 1990s, voluntary counseling and testing services, implemented largely in public health facilities, became the backbone of HIV prevention (4). Other HIV prevention strategies that followed included the expansion of prevention of mother-to-child transmission and voluntary medical male circumcision programs (5). In 2004, antiretroviral therapy (ART) was introduced in South Africa and has since become a crucial tool for HIV prevention. Since then, the South African government has offered ART at no cost through public health facilities nationwide (6).

Oral Pre-exposure prophylaxis (PrEP) reduces HIV transmission risk through sex by nearly 99% when taken as prescribed and is one of the most effective biomedical methods in the HIV prevention toolkit (7). In 2015, oral PrEP was approved for use in South Africa (8), and in 2016, South Africa initiated a phased national program to scale oral PrEP, starting with female sex workers (FSW) (9, 10). Since then, PrEP eligibility has broadened to include other individuals or groups who have a significantly higher likelihood of acquiring HIV due to specific behavioral, biological, or social factors. These include but are not limited to key populations, adolescent girls and young women (AGYW), serodiscordant couples and more recently, pregnant and breastfeeding women (11). The effectiveness of oral PrEP requires adherence to daily pill-taking, which may present challenges for populations who are vulnerable to HIV acquisition, including AGYW. These same populations may experience structural issues related to adhering to oral PrEP including stigma or lack of privacy (12).

While the South African PrEP program has focused on oral PrEP to date, injectable PrEP options, such as long-acting cabotegravir (CAB-LA) and lenacapavir, may offer solutions to the challenges of oral PrEP (13, 14). CAB-LA, administered once every two months and approved for use in South Africa by the South African Health Products Regulatory Authority (SAHPRA) in December 2022, reduces the need for daily medication and can provide discreet, long-lasting HIV prevention (15). Clinical trials have shown this method to be highly effective. In a meta-analysis of clinical trial results, there was a 79% reduction in HIV risk among African women when comparing CAB-LA to oral PrEP (16). Despite its strengths, injectable PrEP also brings with it challenges. In addition to concerns around cost and risk of HIV drug resistance for individuals starting CAB-LA while acutely infected or those who experience a breakthrough infection (16), CAB-LA injections must be given intramuscularly by trained providers in clinical settings which may pose scale-up and access barriers. Innovative delivery methods, such as neighborhood “shot clinics” or mobile health services that can offer access to more convenient injection locations may be needed to not only improve access to injectable PrEP, once introduced at market, but decongest health facilities (17). The dapivirine ring, another long-acting PrEP method, provides additional choice in PrEP options and was approved for use by SAHPRA in March 2022. While vaginal rings are a highly acceptable mode of medication distribution designed to give a regulated and prolonged release of the drug, unlike CAB-LA, the dapivirine ring has low efficacy, reducing HIV acquisition risk by 35%–50%, among other limitations (18, 19).

The Joint United Nations Programme on HIV/AIDS (UNAIDS) has committed to eliminate AIDS as a public health threat by 2030. However, progress has been insufficient, with over 1.5 million new HIV infections in 2020 globally, compared to the objective of 500,000 (20). Global HIV prevention targets aim to have 95% of people at risk of HIV infection use effective combination prevention and less than 370,000 annual new HIV infections by 2025. These fast-approaching targets necessitate a combination of effective prevention options that are uniquely tailored for different individuals and population groups at risk of HIV (21). To achieve these targets and close remaining gaps, we must apply lessons learned and global “best practices” to expand access to HIV prevention.

South Africa has led the world in oral PrEP implementation. Between 2016 and 2023, there were over 5.6 million oral PrEP initiations globally. Of these, over 1.2 million occurred in South Africa, with nearly 700,000 implemented through USAID/South Africa's U.S. President's Emergency Plan for AIDS Relief (PEPFAR) program (22). In this case study, we apply the World Health Organization's (WHO) Building Blocks for Health Systems Strengthening as a framework to describe USAID's oral PrEP program in South Africa–one of the largest PrEP programs in the world–as well as characterize innovative strategies used by USAID and its implementing partners to accelerate access in the country. We report experiences and lessons learnt in the process of introducing and scaling oral PrEP implementation in USAID-supported facilities and communities across 14 districts in 7 provinces. Sharing best practices and lessons learned, as done in this case study, can not only strengthen the implementation evidence base but can inform more efficient PrEP service delivery, particularly as new PrEP products come to market.

## 2 Context

**Figure 1:**
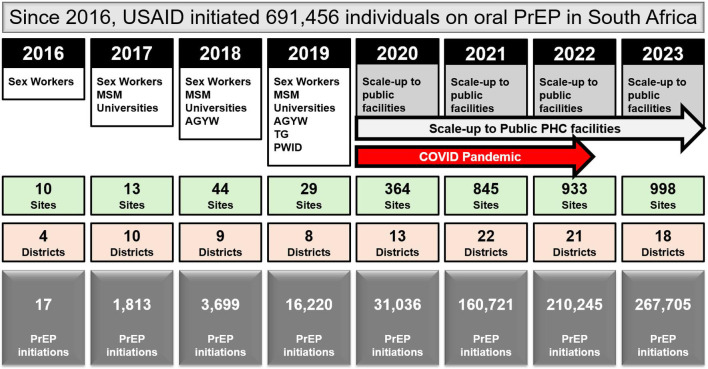
USAID-initiated PrEP rollout in South Africa (2016–2023), showing the expansion of target populations, number of sites and districts, and total PrEP initiations.

**Figure 2:**
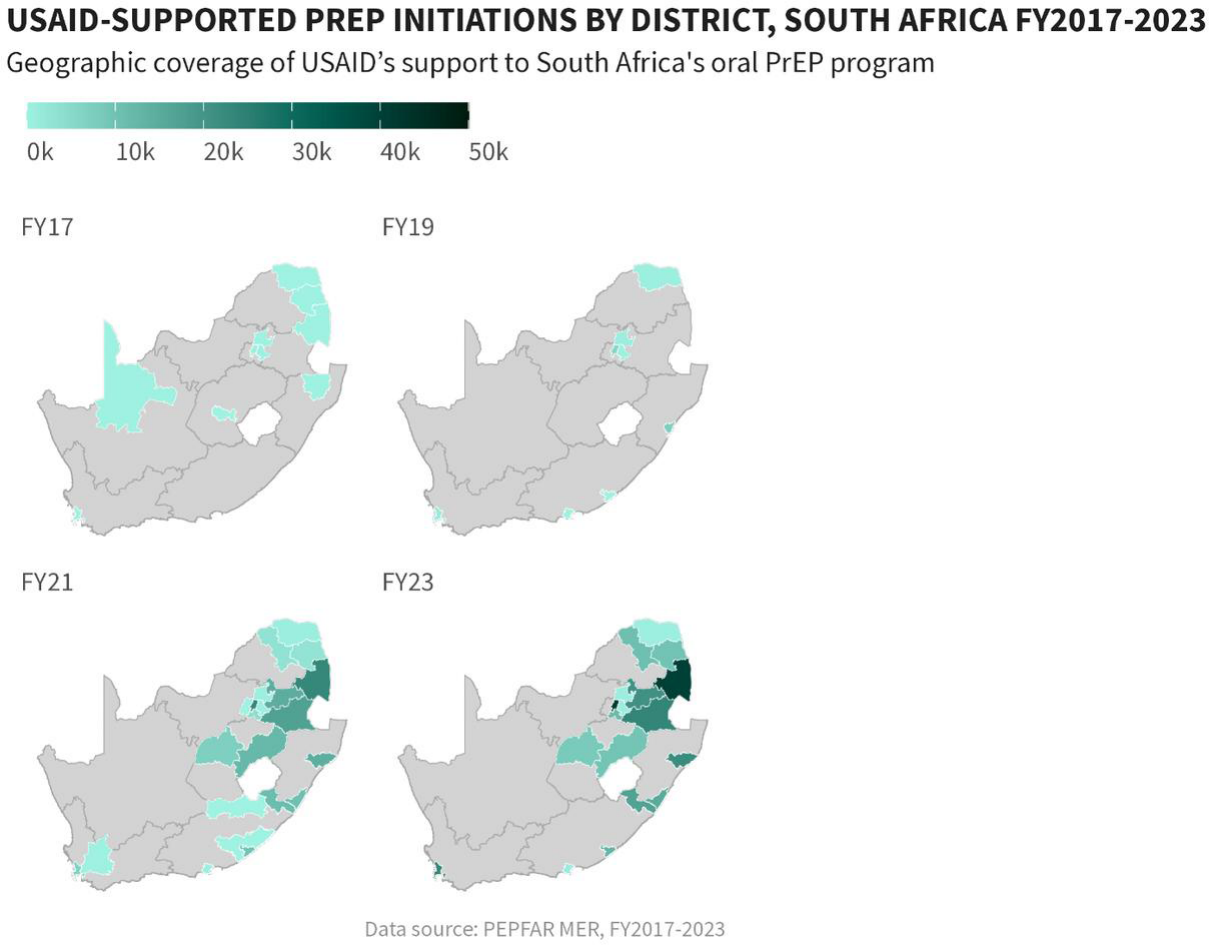
Geographic coverage of USAID-supported oral PrEP initiations by district in South Africa, October 2017–September 2023.

**Figure 3:**
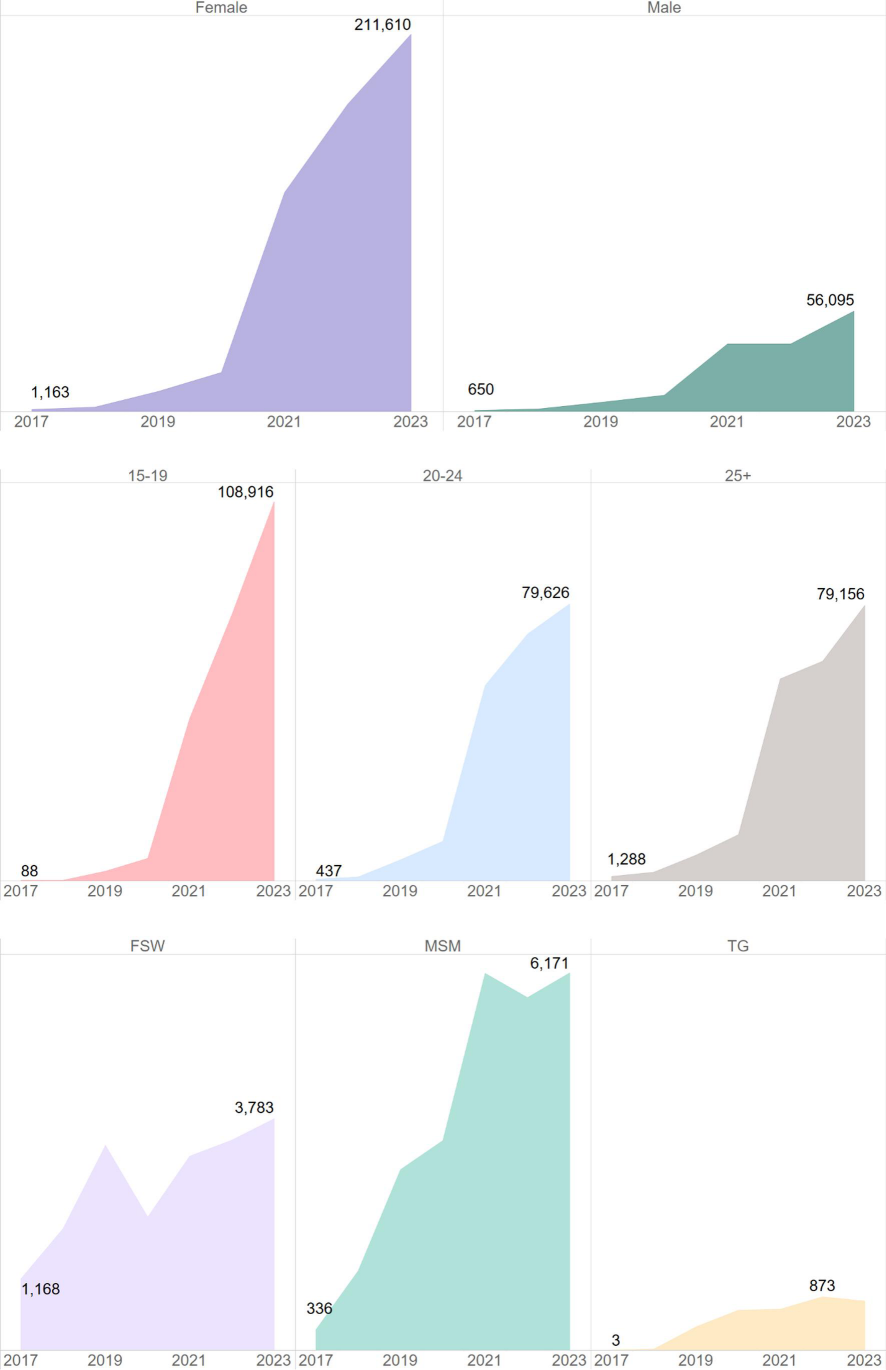
Significant increase in PrEP initiations across various demographic groups, particularly among females and youth 15–19 years, South Africa, October 2017–September 2023.

USAID has supported the oral PrEP program in South Africa since 2016 (23). Timelines and processes for oral PrEP rollout by the Department of Health (DOH) and USAID are provided in Figure 1. Population prioritization for USAID's support aligned to DOH policy, with key populations and AGYW prioritized in 2017/2018 and subsequent scale-up to public health facilities and the general population in 2020. From October 2017 to September 2023, the number of facilities providing oral PrEP with USAID support increased from 10 to 998. In this period, USAID increased implementation support for oral PrEP from four districts in two provinces to eighteen districts across seven provinces. Current and historic geographic coverage for USAID's support to the oral PrEP program is provided in Figure 2. From October 2017 to September 2023, USAID saw an increase in annual PrEP initiations from under 1,000 to over 200,000 among females (Figure 3). Annual initiations among males increased from 600 to 56,000 in the same period. By age group, youth 15–19 years had the highest number of annual initiations, increasing from less than 100 in PEPFAR's fiscal year (FY) 2017 to 109,000 in FY2023. Annual initiations among young people 20–24 years also increased from under 1,000 in FY2017 to nearly 80,000 in FY2023 (Figure 3). Key populations groups also saw a significant increase in PrEP uptake (Figure 3); USAID-supported annual PrEP initiations among men who have sex with men (MSM) and FSW peaked between October 2022 and September 2023 at 6,000 and 4,000, respectively. Across USAID-supported districts from October 2022 to September 2023, there was exceptional achievement in new PrEP initiations with 14 out of the 18 USAID-supported districts reporting an achievement of 100% or higher against annual PEPFAR targets (Supplementary Table S1).

## 3 Applying WHO's six building blocks of health system strengthening to the PrEP program in South Africa

The expansion of oral PrEP in South Africa exemplifies a successful implementation of the World Health Organization's six building blocks of health system strengthening. Here we discuss the application of these building blocks to the USAID-supported PrEP program in South Africa, highlighting key achievements and areas of impact.

### 3.1 Leadership and governance

The leadership and governance of the PrEP program in South Africa are characterized by strong political commitment and strategic direction from the South African government and the National and Provincial DOH. Collaborative efforts between the government, developmental partners, civil society, and the private sector have contributed to a well-coordinated prevention response (24). The South African government's active role in policy development, particularly the inclusion of PrEP in national HIV prevention strategies, has been instrumental in broadening the reach of the program to diverse at-risk populations, such as AGYW (25). A consultative process between the South African government and stakeholders led to the launch of the oral PrEP program in 2016, with FSW prioritized for PrEP services. Through continuous engagement between government and stakeholders, there was agreement to expand PrEP access to include AGYW and in 2017 AGYW started accessing PrEP through development partners (23). By 2020, an agreement among stakeholders was reached to support roll-out of PrEP to include the general population and by 2021, PrEP was accessible through various modalities including at community sites (26). USAID has continued to work closely with the DOH for capacity building and support of human resources for health through direct and indirect service delivery (26). DOH has been an instrumental partner for the successful roll-out of PrEP. The interim reporting systems were developed by DOH which were by 2020 integrated into routine reporting through TIER.Net and District Health Information System (DHIS) (24). The PrEP technical working group steered by DoH provided a multistakeholder coordinating mechanism for sharing early lessons from implementation science studies, cost effectiveness studies and implementation innovations that informed the programmatic scale-up of oral PrEP (24).

### 3.2 Service delivery

**Figure 4:**
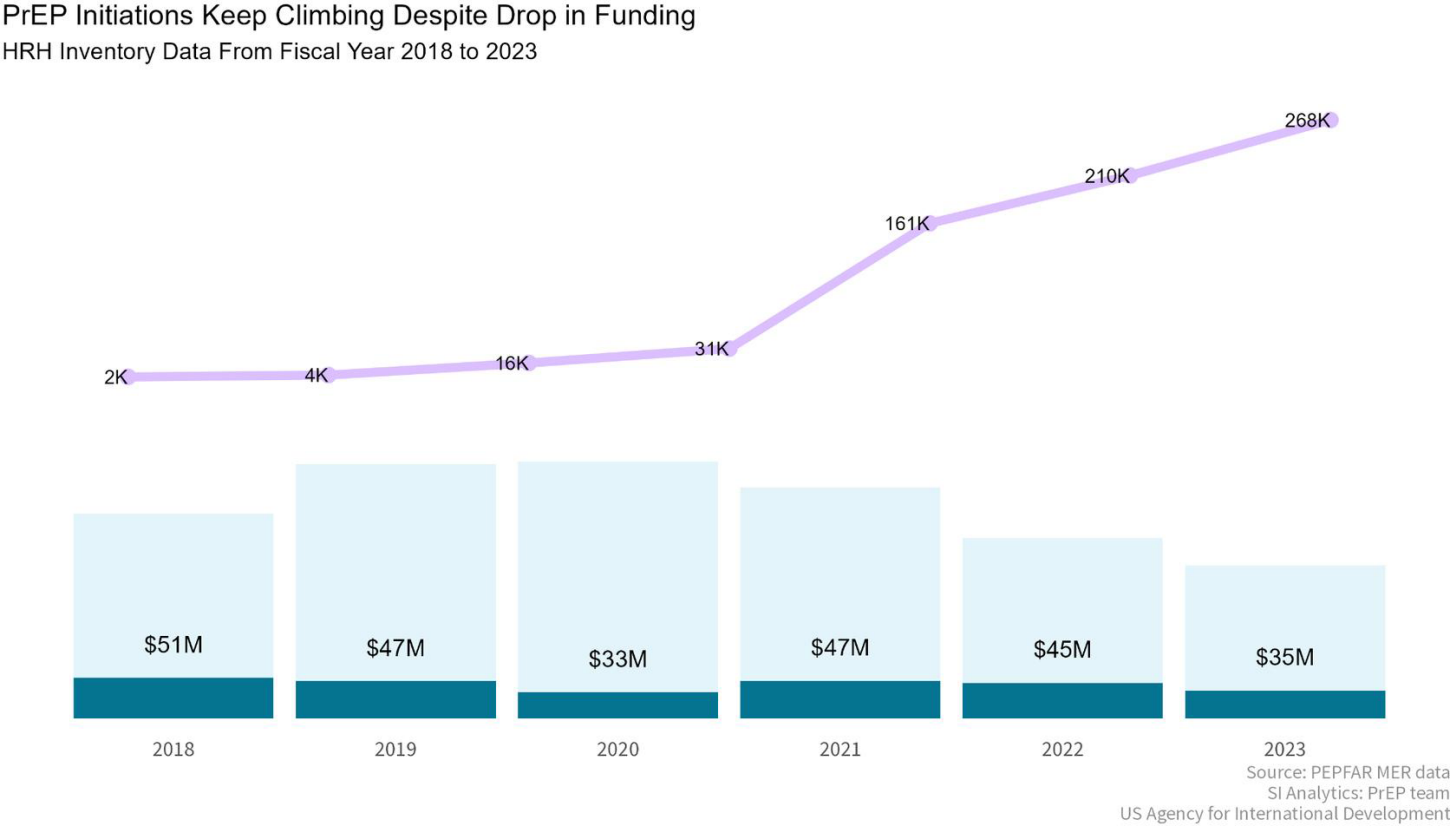
Despite fluctuations in funding, USAID-supported PrEP initiations continue to rise: a comparison of human resources for health inventory data, South Africa, October 2022–September 2023. *Data were not available prior to 2018.

Service delivery is a critical component of the PrEP program's success. The program has prioritized comprehensive services that address the preventative, curative, and health promotion needs of key and priority populations (27). Accessibility has been significantly enhanced through innovative delivery models such as mobile clinics, gazebo-based models, community-led services, and integration within existing health services like sexual and reproductive health and school health services (27). These approaches reduce barriers related to cost, location, and cultural factors, making PrEP more accessible to those in need. Community engagement has also been pivotal, with peer educators from key populations and DREAMS Ambassadors for AGYW playing a crucial role in service delivery, ensuring that services are not only accessible but also acceptable to the target populations (28). The focus on quality and continuity of care ensures that individuals receive consistent and high-quality services across different health conditions and care levels (28). Good service delivery approaches are those which deliver comprehensive, coordinated, safe, quality person-centered health interventions that are accessible, acceptable and responsive to the needs of service beneficiaries and rendered with minimum waste of resources (26). USAID employs a multi-pronged approach for delivery of HIV prevention services focusing on support for the DOH at facility and community levels, through mobile clinics, outreach approaches, and fixed specialized sites for key populations (29). At facility level, direct service delivery and technical assistance is provided to ensure augmentation and capacity building and support of health care workers and to accelerate provision of quality HIV prevention services as well as data quality and data management (30). An enhanced capacity building intervention is provided at the district, provincial and national level to ensure improved leadership, management and implementation of HIV prevention policies and create an enabling environment for health system and policy implementation (24). Community-based PrEP, a key focus for USAID in recent years to accelerate access to PrEP, followed a rigorous process to ensure acceptability and accessibility of PrEP as part of the comprehensive sexual and reproductive services (24). Engagements and consultations happened at various levels which included civil society organizations, South African government, community leaders, healthcare workers as well as service beneficiaries to ensure PrEP is accepted as an additional HIV prevention method and service delivery quality and requirements are maintained when implementing PrEP services in communities (24). USAID collaborated with regional training centers to include PrEP training in annual training plans, and cascaded training and mentoring and support for in-facility PrEP service delivery as oral PrEP was rolled out (26). Additionally, efforts were made to build and maintain a service delivery model that is resilient and capable of responding to the impact of broader health system shocks and aftershocks, such as those experienced during the COVID-19 pandemic, demonstrated by the sustained increased in USAID-supported PrEP initiations after 2020 (Figure 4).

### 3.3 Health workforce

**Figure 5:**
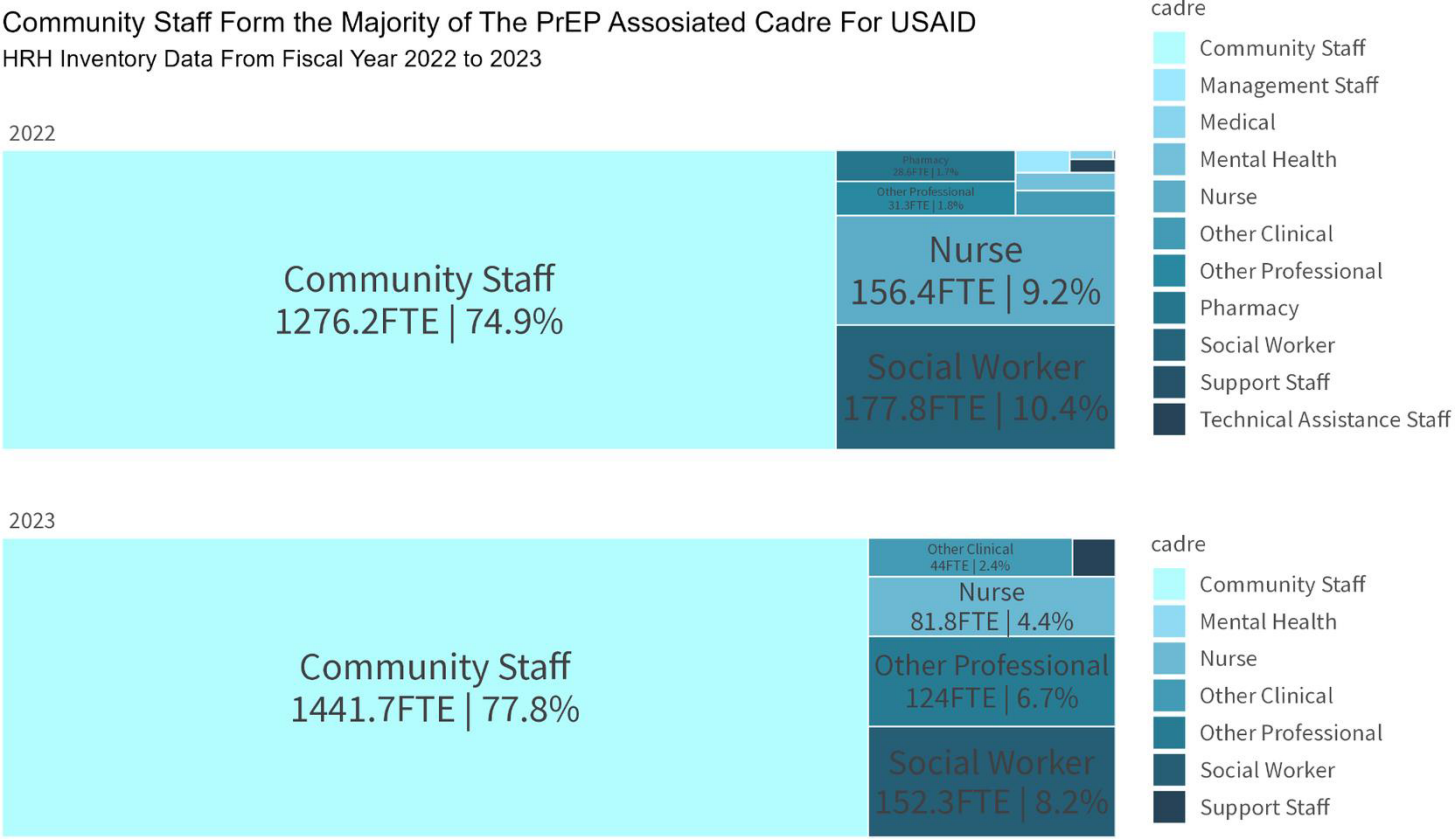
USAID human resources for health inventory data on PrEP implementation, South Africa, October 2022–September 2023.

The effectiveness of USAID's PrEP program has relied on a well-trained and motivated health workforce. Capacity building initiatives have been central to the program, with extensive training and support provided to professional nurses, case managers, and lay cadres (31). These initiatives include task-shifting and task-sharing approaches, which have expanded the program's capacity to deliver PrEP services and provide necessary psychosocial support. The involvement of PrEP Ambassadors and Determined, Resilient, Empowered, AIDS-free, Mentored, and Safe (DREAMS) Ambassadors has proven particularly effective in supporting AGYW with adherence to and continuation of PrEP, addressing the unique challenges faced by this demographic (31). Equally, involving peers in outreach services and mobilization has enabled key populations to feel comfortable discussing concerns and challenges around PrEP uptake and use, and feel supported in their PrEP journey (32). The remarkable performance of the USAID PrEP program was spearheaded by a human resources for health cadre that included a broad spectrum of roles split between health facilities and the community. More than 75% of cadres supporting USAID's oral PrEP program were community based in FY2022 and FY2023 (Figure 5).

### 3.4 Health information systems

Robust health information systems are essential for the effective monitoring and evaluation (M&E) of the PrEP program (33). The introduction of oral PrEP required development of clinical stationary and national M&E tools. Modifications to the electronic patient monitoring data system, Tier.net, as well as the aggregate national District Health Information Software 2 (DHIS2) followed. USAID implementing partners developed interim M&E tools to capture and track oral PrEP initiations and use for PEPFAR reporting purposes while modifications to national systems were implemented (24, 34). Since modifications to national systems were made, USAID partners have leveraged Tier.net as the data source for reporting on oral PrEP. These systems ensure that program planning and evaluation are informed by accurate and timely data, enabling continuous improvement of PrEP services (35, 36).

Leveraging lessons learned from the introduction of oral PrEP and in anticipation of new PrEP products being scaled up, the National DOH made modifications to PrEP clinical stationary to allow for implementation science studies to report on injectable cabotegravir and the dapivirine ring (37).

As of 2024, the NDOH is developing a national electronic health record. This will replace Tier.Net in monitoring PrEP uptake (38). With the roll-out of new PrEP products through implementation science studies in 2022, USAID partners now use the revised national paper-based stationery to collect data on new PrEP products and enter information into their partner information systems (39).

### 3.5 Access to essential medicines

Ensuring equitable and continued access to PrEP products has been fundamental to the oral PrEP program in South Africa. The program began as a donor funded program, including a substantial donation from Gilead Sciences, as well as PrEP product procured by PEPFAR in South Africa, until 2020 when the DOH began managing procurement of PrEP drugs directly and implementing service delivery at public health facilities (9, 10). By 2023, oral PrEP was fully procured by DOH and USAID implementing partners could access PrEP through the district pharmacies. Throughout this period, effective supply chain management practices, supported in part by USAID partners at various levels, have been implemented to guarantee the availability of PrEP medicines (31).

This included support to address constraints and improve availability of medicine, a core objective of South Africa's NDoH. Our Implementing Partners worked with the Department to design and implement innovative solutions to improve access to and availability of PrEP medicines through accurate forecasting, demand and supply planning of PrEP medicines (31). Simplified branding and packaging of PrEP medicines have also been recommended to reduce stigma and improve uptake and continued use. Quality assurance measures, including pharmacovigilance, ensure the safety and efficacy of PrEP products, further supporting their widespread adoption (40). To promote PrEP choice and generate early evidence to inform the rollout of new PrEP products, USAID prioritized implementation science activities for new HIV prevention methods, including CAB-LA and the dapivirine ring, soon after their regulatory approvals (41). Among several implementation science studies in South Africa, USAID's DREAMS PrEP Choice Study is a mixed methods demonstration project examining the acceptability and feasibility of introducing the dapivirine ring alongside oral PrEP in community settings under the DREAMS program (22). The study looks at differentiated service delivery models and includes a prospective observational cohort as well as qualitative methods. Catalyzing Access to New Prevention Products to Stop HIV (CATALYST), a multi-country study supported by USAID, aims to describe the implementation of an enhanced service delivery package for PrEP choice across three approved PrEP products (oral PrEP, dapivirine ring, and CAB-LA) among women in several countries, including South Africa. CATALYST consists of a prospective cohort, a process evaluation, and several nested studies to better understand acceptability, adherence, costing, and medicine resistance (22).

### 3.6 Health financing

Long term sustainability of the PrEP program relies on robust health financing mechanisms. Between October 2018 and September 2023, the average annual funding contribution for USAID-supported PrEP services was $43 million, demonstrating a significant investment in HIV prevention. Despite fluctuations in funding levels over the period, the program has continued to expand, with PrEP initiations increasing even during periods of financial constraints (Figure 4). The program's financial accountability is maintained through efficient and transparent allocation of resources, ensuring that funds are used effectively to support and expand PrEP services. Innovative financing approaches, such as strategic public-private partnerships (42), are being explored to supplement traditional funding sources. These initiatives aim to ensure the long-term sustainability of the program (24).

## 4 Discussion

### 4.1 Practical implications and lessons learned

Strong political commitment and strategic governance are critical for the successful implementation and scale-up of health programs like oral PrEP in South Africa (43). Active involvement and collaboration across various stakeholders, including government bodies, donor organizations, and the private sector, enhance resource mobilization and program sustainability. Establishing clear policy frameworks and maintaining political will foster ownership and accountability, essential for long-term success. Innovative service delivery models, such as mobile clinics and community-led services, increase accessibility and acceptability, particularly for marginalized populations (24). Integrating PrEP services with other health services, including sexual and reproductive health services, improves efficiency and coverage and decreases stigma, while ongoing community engagement, and use of peer educators/champions/ambassadors significantly boost service uptake and continued use (44). Investing in the training and capacity building of healthcare workers, including nurses, case managers, and lay cadres, ensures a competent workforce capable of delivering high-quality PrEP services (45). Task-shifting, task-sharing, and peer-led approaches enhance service delivery and support, addressing unique challenges faced by specific populations. Robust and agile health information systems are vital for effective program M&E, facilitating informed decision-making and continuous quality improvement (45). Ensuring equitable access to PrEP through efficient procurement, flexible delivery models, and user-friendly packaging reduces stigma and improves uptake. Sustainable financing mechanisms and transparent resource allocation are crucial for ensuring program continuity and expansion, with innovative financing approaches supplementing traditional funding sources to ensure long-term viability (46). A significant lesson from the PrEP rollout in South Africa was that introducing PrEP initially among FSW inadvertently contributed to additional stigma, as the drug became perceived as a “sex worker drug.” This underscores the necessity for careful planning and implementation when introducing new HIV biomedical prevention, emphasizing the importance of minimizing stigma both among key populations and in the broader context of HIV prevention and treatment (27).

### 4.2 Introduction of new PrEP products

The development of new PrEP products, such as injectable cabotegravir, the dapivirine ring, and lenacapavir, represents a significant advancement in HIV prevention (47). These products offer additional options for individuals facing challenges with daily oral PrEP adherence. Introducing and implementing new products as part of routine service delivery not only requires regulatory approval, but integration into existing supply chain and health information systems, healthcare worker training, and community engagement activities (48).

## 5 Limitations

With the exception of the DREAMS program, PrEP data are reported at aggregate level. Aggregate data, while useful for high-level overviews, lacks the granularity needed for detailed individual-level analysis. The top-level aggregations are by age and sex. This limitation can obscure important variations and trends within sub-populations, making it difficult to identify specific areas needing targeted interventions. Although oral PrEP data are typically reported with age and sex disaggregates, the absence of patient-level data limits the capacity to accurately represent and adequately target key populations, including men who have sex with men (MSM) and female sex workers (FSW).

Additionally, this lack of detailed data hinders the ability to effectively monitor PrEP adherence, thereby compromising the assessment of its effectiveness within these high-risk groups. The variability in data quality and reporting standards due to the transition from multiple implementing partners to a centralized reporting system by the National DOH presents another challenge. Different partners might have employed varying methodologies and standards of reporting, leading to discrepancies in the data. Furthermore, delays in data availability can impede timely decision-making and the ability to respond swiftly to emerging trends or issues within the program. Timely data are essential for proactive management and adaptation of HIV prevention strategies.

Integrating data from multiple sources, such as Tier.net and PEPFAR's system, is complex and prone to errors, which can lead to inconsistencies and gaps in the dataset. There is also a potential for data duplication, especially if there were overlaps or redundancies in the data reported by different entities, or in the absence of a unique patient identifier.

Addressing these limitations requires efforts to standardize data collection and reporting practices, improve data integration systems, ensure timely availability of comprehensive data, and enhance the granularity and representation of key populations in the data. Continuous M&E, coupled with adaptive management strategies, are essential to mitigate these challenges and optimize the effectiveness of HIV prevention programs.

## 6 Conclusion

The scale-up of South Africa's PrEP program, underpinned by USAID and PEPFAR support, exemplifies a highly coordinated response to the HIV epidemic, successfully applying WHO's Six Building Blocks of Health Systems Strengthening. This case study offers valuable insights into the multifaceted requirements for effective HIV prevention, including the need for sustained political commitment, strategic governance, comprehensive service delivery, a skilled and motivated health workforce, robust health information systems, and innovative financing solutions. Despite significant progress, challenges remain, particularly in reducing stigma and ensuring equitable access across diverse populations.

The introduction of long-acting PrEP products, such as CAB-LA the dapivirine vaginal ring and lenacapavir, presents new opportunities to address adherence barriers associated with daily oral PrEP, potentially expanding reach to populations previously underserved. However, integrating these new products requires careful planning to navigate operational complexities, including regulatory processes, healthcare worker training, and community acceptance. Ultimately, to meet global HIV prevention targets, a flexible, evidence-based approach that adapts to emerging needs and incorporates novel prevention tools will be essential. This case study underscores the importance of a responsive, resilient PrEP program that is adaptable to future innovations and can serve as a model for other high-burden settings aiming to eliminate HIV as a public health threat.

## Data Availability

Publicly available datasets were analyzed in this study. This data can be found here: https://data.pepfar.gov/datasets.

## Supplementary material

Supplementary Table S1 Target achievement for the number of new PrEP initiations (PrEP_NEW) across districts support by PEPFAR through USAID, South Africa, October 2022–September 2023.
