# Corrigendum: Oral pre-exposure prophylaxis implementation in South Africa: a case study of USAID-supported programs

**DOI:** 10.3389/frph.2025.1560111

**Published:** 2025-01-24

**Authors:** Jerome Wendoh Milimu, Lauren Parmley, Mahlodi Matjeng, Mathata Madibane, Mandisi Mabika, Jacques Livingston, Joseph Lawrence, Orapeleng Motlhaoleng, Hasina Subedar, Rethabile Tsekoa, Zandile Mthembu

**Affiliations:** ^1^ Bilateral Health Office, United States Agency for International Development, Pretoria, South Africa; ^2^ National Department of Health, South African Government, Pretoria, South Africa

**Keywords:** HIV prevention, pre-exposure prophylaxis, PEPFAR program, South Africa, cabotegravir, dapivirine ring, lenacapavir, health systems strengthening

A Corrigendum on Oral pre-exposure prophylaxis implementation in South Africa: a case study of USAID-supported programs By Milimu JW, Parmley L, Matjeng M, Madibane M, Mabika M, Livington J, Lawrence J, Motlhaoleng O, Subedar H, Tsekoa R and Mthembu Z (2024). Front. Reprod. Health 6:1473354. doi: 10.3389/frph.2024.1473354

**Text Correction**

In the published article, there was an error. [“Specifically, in the introduction, there is a reference to the South African Health Products Regulatory Authority, which has been abbreviated twice as “SAPHRA.” However, the correct abbreviation is “SAHPRA.”].

A correction has been made to **[Introduction]**, [Paragraph Numbers 69 & 81]. This sentence previously stated:

“[SAPHRA]”

The corrected sentence appears below:

“[SAHPRA]”

The authors apologize for this error and state that this does not change the scientific conclusions of the article in any way. The original article has been updated.

**Request to add ORCID identifiers for two authors**

The first and second authors would like to request that their ORCiD identifiers be linked to the published article. The respective names and ORCiD identifiers are as shown below.

Jerome Wendoh Milimu (0000-0002-3383-2087)

Lauren Parmley (0000-0001-5119-5811)

The authors apologize for this error and state that this does not change the scientific conclusions of the article in any way. The original article has been updated.

