# Corrigendum: Oral pre-exposure prophylaxis implementation in South Africa: a case study of USAID-supported programs

**DOI:** 10.3389/frph.2025.1561341

**Published:** 2025-01-27

**Authors:** Jerome Wendoh Milimu, Lauren Parmley, Mahlodi Matjeng, Mathata Madibane, Mandisi Mabika, Jacques Livingston, Joseph Lawrence, Orapeleng Motlhaoleng, Hasina Subedar, Rethabile Tsekoa, Zandile Mthembu

**Affiliations:** ^1^ Bilateral Health Office, United States Agency for International Development, Pretoria, South Africa; ^2^ National Department of Health, South African Government, Pretoria, South Africa

**Keywords:** HIV prevention, pre-exposure prophylaxis, PEPFAR program, South Africa, cabotegravir, dapivirine ring, lenacapavir, health systems

A Corrigendum on Oral pre-exposure prophylaxis implementation in South Africa: a case study of USAID-supported programs By Milimu JW, Parmley L, Matjeng M, Madibane M, Mabika M, Livington J, Lawrence J, Motlhaoleng O, Subedar H, Tsekoa R and Mthembu Z (2024). Front. Reprod. Health 6:1473354. doi: 10.3389/frph.2024.1473354

**Incorrect author name**

Please also check that the initials used in the Author Contributions section or elsewhere in the article are correct.

In the published article, an author name was incorrectly written as [Jacques Livington]. The correct spelling is [Jacques Livingston].

The authors apologize for this error and state that this does not change the scientific conclusions of the article in any way. The original article has been updated.

