# Supplementary material for: Oral pre-exposure prophylaxis implementation in South Africa: a case study of USAID-supported programs
Source: Front Reprod Health. 2024 Dec 10;6:1473354. doi: 10.3389/frph.2024.1473354 (PMC11666530; doi:10.3389/frph.2024.1473354)
Supplement: Supplementary Table S1 — Target achievement for the number of new PrEP initiations (PrEP_NEW) across districts support by PEPFAR through USAID, South Africa, October 2022–September 2023. [file Table1.docx]

| PrEP Initiations Target Achievement by USAID-Supported District | | | |
| --- | --- | --- | --- |
| October 2022-September 2023 Results | | | |
| District | New PrEP initiation results | New PrEP initiation targets | Target Achievement |
| **Mpumalanga** |  |  |  |
| Ehlanzeni | 39904 | 14330 | 278% |
| Gert Sibande | 23600 | 9270 | 255% |
| Nkangala | 20713 | 12180 | 170% |
| **KwaZulu-Natal** |  |  |  |
| King Cetshwayo | 21931 | 8310 | 264% |
| Ugu | 15094 | 6790 | 222% |
| **Eastern Cape** |  |  |  |
| Alfred Nzo | 16930 | 8480 | 200% |
| Buffalo City | 12697 | 6793 | 187% |
| Nelson Mandela Bay | 944 | 953 | 99% |
| **Free State** |  |  |  |
| Lejweleputswa | 8195 | 5130 | 160% |
| Thabo Mofutsanyane | 9370 | 6390 | 147% |
| **Gauteng** |  |  |  |
| Sedibeng | 11668 | 7730 | 151% |
| City of Johannesburg | 41172 | 28673 | 144% |
| City of Tshwane | 706 | 832 | 85% |
| Ekurhuleni | 701 | 860 | 82% |
| **Limpopo** |  |  |  |
| Vhembe | 560 | 440 | 127% |
| Capricorn | 10519 | 10440 | 101% |
| Mopani | 9531 | 9490 | 100% |
| **Western Cape** |  |  |  |
| City of Cape Town | 23385 | 25482 | 92% |
| Source: PEPFAR MER Data | | | |
